# Integration of advanced practice providers into the Israeli healthcare system

**DOI:** 10.1186/s13584-016-0065-8

**Published:** 2016-02-22

**Authors:** Eliana Marcus Aaron, Caryn Scheinberg Andrews

**Affiliations:** Sheba Medical Center at Tel Hashomer, Ramat Gan, Israel; Henrietta Szold School of Nursing, Hadassah Medical Organization, Hebrew University, POB 12000, Ein Kerem, Jerusalem Israel

**Keywords:** Nurse practitioners, Physician assistants, Advanced practice nurses, Israel health, Nursing role development, Physician shortage, Nursing health policy, Healthcare access, Certified registered nurse anesthetist, International nursing, Nurse migration

## Abstract

Many countries around the world have integrated various types of Advanced Practice Providers (APPs) into their healthcare systems. The main motivating factors for recognizing and developing APPs worldwide include physician shortages and the need for improved access or delivery (US, France, Belgium, Scotland, Switzerland), reduced residency hours (US, UK), shortages in underserved regions (US, Canada, Finland, Australia), and cost containment (Germany, Netherlands, UK, US).

Israel is experiencing a shortage of physicians in peripheral geographic regions and in critical medical specialties. Recent by-laws approved by the Knesset (Parliament), combined with Israel Ministry of Health (MOH) policies, have thus far been unable to fully address the shortages. To understand the potential contribution of APPs in Israel, we evaluated the international historical foundations and development of APP roles. We assessed how APPs have impacted healthcare in other countries by analyzing public data and published international research about APP education, safety, quality of care, motivators, barriers, and impact. We found that APPs are recognized in dozens of countries, and have similar scopes of practice, graduate level education requirements (in developed countries), and clinical training.

At the same time, there is wide variability among countries in the actual function and independence of the advanced practice nurse (APN), particularly the nurse practitioner (NP). APPs have been established as cost effective, safe healthcare providers who improve healthcare access.

Israel has begun to introduce APPs, specifically NPs, in a variety of fields, including geriatrics, palliative care and diabetic care. We recommend a rapid expansion of existing and new APP roles into the Israeli healthcare system based on evidence and the recommendations of international evaluations by non-government organizations. By shifting the education to a university setting, mirroring successful, evidence-based, and established APP models found internationally, Israel could lessen the projected Israeli physician shortage, improve healthcare access in specific areas, and bolster existing resources towards a larger and richer pool of healthcare providers in Israel.

## Introduction

The ratio of working Israeli physicians to the population has been decreasing for years due to a decline in immigration rates, retirement of a large cohort of physicians, and attrition. At the same time, the need for medical services has increased due to both the population growth (high birth rate) and an increasingly aging population [1–3]. Attrition includes physicians who emigrate to other countries for economic reasons and better working conditions (“brain drain”), or leaving clinical practice for other jobs, such as research and development in the pharmaceutical or biotech industries [4, 5]. Moreover, burnout rates among Israeli physicians are also reported to be high [6] which results in physicians leaving clinical practice or the country [7]. Overall, nationally, there are physician and resident positions that remain unfilled [8].

Actual or predicted physician shortages are not only an Israeli problem, but are also a worldwide problem. This has led many governments and international organizations to seek solutions for providing adequate healthcare services to expanding, aging populations. The World Health Organization (WHO), Institute of Medicine (IOM), Organisation for Economic Co-operation and Development (OECD), and the US Agency for Healthcare Quality Research (AHQR) have been studying the utilization and effect of adding non-physician healthcare providers, such as advanced practice nurses (APNs) in most cases nurse practitioners (NPs), into the mix of healthcare providers, in order to supplement strained physician-only systems. Results of these studies have been promising, showing that care provided by both physician and non-physician providers is equivalent, safe, cost-effective, and produces high levels of patient satisfaction [9–15]. Reports from North America, Europe, Asia, and Oceania reflect similar outcomes with the addition of APNs into healthcare settings that are suffering from physician shortages [16].

In Israel, the Knesset (Parliament) recently approved by-laws establishing the NP profession in one effort to alleviate a projected shortage of Israeli physicians relative to the OECD average [17]. The newly established non-physician healthcare provider role started as pilot NP programs in geriatrics and palliative care, which are considered to be areas of extreme physician shortages in Israel [18].

In spite of a projected physician shortage, and the approved regulations, Israel has been slow to recognize, develop, and incorporate NPs into the healthcare system. Most importantly, although most healthcare provision in Israel remains community-based, as of now, Israel’s Ministry of Health (MOH) has not recognized or developed NPs for community-based settings. The purpose of this paper is to report on the current status of Israeli healthcare provider shortages, describe an overview of the current state of selected Advanced Practice Providers (APPs) development in the world (specifically nurse practitioners, (NPs), physician assistants (PAs), and certified registered nurse anesthetists (CRNAs), and suggest a framework for modifying current practices in Israel in light of the challenges facing Israeli healthcare and based on international literature and experience.

Specific aims include:

- Describing the physician and nursing shortages in Israel
- Discussing Israel’s solutions to the shortages
- Defining and describing the roles, education, impact, and recognition of APPs
- Reviewing evidence regarding safety and quality of care provided by APPs
- Motivating factors to APP implementation internationally
- Barriers to APP implementation internationally
- Status of APPs in Israel
- Discussing health policy implications of APPs in Israel
- Specific recommendations for APP integration in Israel.

## Israeli physician and nursing shortages

According to official Ministry of Health statistics, Israel's ratio of physicians age 65 or younger, to the total population, decreased from 3.4 in 2005 to 3.1 in 2014 [19]. Whereas Israel once boasted one of the world's highest physician to population ratios, the latest comparative data indicate that Israel's ratio of practicing physicians to 1,000 population (3.4) is very similar to that of the Organization for Economic Co-operation and Development (OECD) average (3.3) and that the Israel-OECD gap has narrowed substantially over time [20]. Predictions of shortages have been made since the early 2000s, with the latest MOH projections suggesting that the ratio noted above is expected to stabilize at 3.0. A 2010 Israeli MOH report (2010) presented similar findings [21, 22], while setting 2.9 as the minimal acceptable level for the number of working age physicians per 1,000 population.

The Israeli Medical Association (IMA) states that an adjusted physician ratio is necessary given the realities in the Israeli healthcare setting. In their calculation, the ratio as of 2010 is reduced to 2.8 practicing physicians per 1000 population, among the lowest level of developed nations [5]. The present and progressive physician shortage in Israel has been a top national priority for its MOH. As seen in other countries, overworked, understaffed health professionals contribute to increases in errors and decreased quality of health services [23, 24].

As of 2012, half of Israel’s physicians were reported by the OECD to be over the age of 55, the highest percentage of older physicians among OECD countries (46 %) [22, 25], and this number has risen to 50 % as of 2014 [20]. With a mandatory physician retirement of 67–70 years old, Israel’s MOH projects critical shortages within 15 years. Even if the present mandatory retirement age of 67 is raised, nearly half of Israel’s physicians will retire in the next 10–20 years^1^. As of 2011, Israel had the lowest medical school graduate rate per capita of any OECD country, or 4.9 graduates per 100,000 residents [26]. While in recent years this has been offset somewhat by Israel's relatively high rate of immigrant physicians and Israelis pursuing medical education abroad (and re-immigrating), the future trajectory of these sources remains an uncertainty, and cannot be relied upon to augment physician ratios.

According to the Israeli MOH, physician shortages can be broken down into geographic scarcities and deficits in specific specialties including geriatrics, anesthesiology, intensive care, surgery, and pediatric subspecialties. There is also an expected shortage of primary care providers working in community health. The shortages are magnified due to the increasingly complex nature of patients (patients with multiple chronic conditions; patients who have had major acute illnesses requiring hospitalization), natural population growth, increased life expectancy, and chronicity of living with managed disease [1, 3]. Table 1 depicts trends of physician ratios in Israel with comparative data from the OECD, reflecting the severity of current and predicted shortages.

**Table 1:** Comparative data: Selected physician ratios and relative growth

Physicians per 1000 residents	
OECD country average 2011	3.1
Top 15 OECD countries 2010	3.5
Israel 1990s	3.5
Israel 2009	3.21
Israel 2011 *Adjusted, practicing physicians (IMA*)	2.8
Israel 2005 periphery –North	2.2
Israel 2005 Tel Aviv/Center	4.7
Israel 2025 (predicted)	2.6
Growth of Population, Physician Ratio	
Growth of Israeli population 2000–2009	18 %
Net growth of physicians 2000–2013	0 %

Sources: [5, 21, 26, 28, 31, 136]

The literature reflects acknowledgement of a severe and growing shortage of anesthesiologists in Israel. This deficit impacts patient access to healthcare by increasing wait times for even semi-urgent surgeries throughout the country, but especially in the periphery where wait times can exceed a year. Operating rooms throughout the country remain under-utilized and patients are often denied evidence-based timely surgical interventions. Consequently, there is a lack of sufficient routine obstetric anesthesia in most hospitals throughout the country [1, 5, 27–30].

Israel also suffers from a significant, growing nursing shortage. Between 2000 and 2013, Israel and the Lithuania were the only two OECD countries to experience a *negative* growth rate in nurses [31]. Israel has one of the lowest nurse-per-population ratios among OECD countries, with 4.8 nurses per 1000 residents, compared to an average of 8.4 nurses in developed countries [25, 26, 32]. According to a 2011 national study, 11 % of Israeli nurses under age 60 did not work as nurses [21], while an Israeli 2014 MOH report shows that 26 % of Israeli nurses do not work in the health sector at all [33], indicating that the nursing attrition trend is increasing. When taking into account nurses who live in Israel and work in the health sector, the nurse ratio is further reduced to 4.2 nurses per 1000 population. Although the number of nursing schools has been steadily increasing in recent years, resulting in an increase in graduate nurses from 11.2 to 15.9 per 100,000 population from 2010 to 2013, Israel remains the country with the second to lowest nurse graduate rate of OECD countries, after Mexico. There is a substantial lag in graduate nurses compared to the rate of nursing retirement and the increasing needs of the population [21, 34, 35].

Israel’s ratio of nurses to physicians is 1.36, compared to an OECD average of 2.79 nurses per doctor [36]. Evidence indicates that Israeli nurses suffer from poor work conditions and practice environments, particularly due to bureaucratic processes for creating needed nursing positions in hospitals [37]. Previous research has shown that poor practice environments contribute to nursing shortages and poor clinical outcomes [38, 39]. Moreover Israel has the most overcrowded hospital system in the developed world, with an average of 98 % occupancy rates in hospitals, compared to 78 % OECD average [26]. Overcrowding in hospitals has been associated with higher rates of adverse events, mortality, hospital acquired infections [40–43], and staff illness rates [44].

Israel’s policy of unionized, nationally regulated nursing wages, as opposed to free market wages, may be harming, rather than helping, nursing and healthcare in Israel, where the average national non-nursing wage is relatively high. International evidence among countries with unionized nursing wages and high non-nursing wages, shows that the unintended consequences of this policy include producing increased dissatisfaction among nurses and even increased patient mortality rates [45]. In addition, Israel nursing staff ratios and “allowed” positions are designated by the Israeli MOH and the national nursing union, further limiting free market in nursing. Hospital-based nurses, consisting of nearly 70 % of professionally active nurses, are simply overworked in Israel’s overcrowded hospitals [33].

In summary, Israel’s health professional shortages, particularly in nursing, are among the most severe in the developed world. Increasing the absolute number of physicians and nurses, as well as the relative number of healthcare providers per capita in a rapidly increasing population, is of utmost importance to solving the shortages. Furthermore, promoting free market conditions may be beneficial to improving the balance of healthcare needs with the growth of healthcare professions in Israel.

## Israel’s solutions to physician and nursing shortages

The Israeli approaches to the physician shortage have mostly focused on increasing the physician workforce. Responses by the Israeli MOH and others, include financially incentivizing Israeli medical residents to work in needed areas, incentivizing foreign physicians to move to Israel^2^, shortening and modifying medical education, creating more medical schools and student slots, improving work conditions [1, 3, 21], and introducing non-physician healthcare providers [46]. In fact, in recent years an emphasis has been placed on enlarging medical school classes nationally and the establishment of a fifth medical school in Northern Israel. Despite these progressive changes, the lengthy training required for physicians means that shortages are expected to persist at least for several years [21, 25]. This is true despite the recent increase in the number of newly licensed physicians, from 715 in 2010 to 1,184 in 2014 [19], which was due to increases both in the number of Israeli-trained physicians and in the number of physicians trained abroad. Moreover, the ratio of working age physicians to population remained essentially unchanged between 2013 and 2014 (at approximately 3.1).

Financial incentives to Israeli medical residents, including monetary advances and bonuses for choosing unpopular specialties or residencies in the understaffed geographic periphery, has thus far been implemented. Early studies suggest that they have been successful in more equitably distributing physician coverage is areas of scarcity (whether geographic or specialty-related) [8].

Bureaucratic impediments have slowed the advancement of some of the other ideas. For example, foreign-trained immigrating physicians historically suffered lengthy procedures and requirements to obtain recognition and licensure in Israel, and as such, are often dissuaded from immigrating or from working as physicians in Israel. Though policy measures to remedy this have been implemented, such as Knesset (Parliament) approval for recognizing the US Medical Licensing Examination (USMLE) [47, 48], the overall effect has yet to be realized. The 18^th^ Israeli Knesset (Parliament) (2009–2013) further reported that some immigrating physicians are leaving Israel due to the bureaucracy in obtaining medical licenses [49]. Moreover, many years will pass before the impact of the proposed solutions will be palpable in Israeli healthcare [1, 21].

In 2013, an Israeli MOH panel evaluated utilizing non-physician medical providers, or APPs, such as NPs and PAs in Israel to alleviate the physician shortage. The report described NP and PA roles, proposed the necessary educational preparation for these two new roles in Israeli healthcare, and determined which role was suited to which need vis-à-vis the Israeli physician shortages [46].

There have been few proposed policy-based solutions to Israeli nursing shortages. Despite evidence of increased nursing schools and nursing graduates in recent years, Israel’s nursing shortage remains a growing problem. There is neither evidence in the literature nor government reports to suggest that policies have been implemented to close the current nursing shortage or to further increase nursing graduate rates. Likewise, processes for integrating immigrant nurses have become more bureaucratic and difficult over the last decade, hence nurse immigrants do not represent a substantial change in the nursing shortage.

In summary, there is an extensive national effort towards increasing physician recruitment, retention, and immigration, as evident by policy development and parliamentary involvement. There is also new legislature approving NPs, which increases healthcare providers in Israel. In contrast, there is no known published, comprehensive national plan to improve the recruitment and retention of nurses in Israel.

## Advanced practice providers

Internationally, the development of APP professions was initiated to increase the number of healthcare providers and improve accessibility to care [50, 51], particularly in peripheral or rural regions [52]. These APP roles include nursing-based direct care providers (APNs) such as NPs, CRNAs, and certified nurse midwives (CNMs), and non-nursing based practitioners such as PAs.

In an OECD analysis of APN roles [9] it was reported that the US, Canada, and the United Kingdom (UK) have the longest experience with APN roles (p.20). As such, the largest body of research on APNs originates from English-speaking countries. The impetus for other countries to develop APN roles stems from the evidence-based data and international evaluations of the successes of APN roles. The US remains the international leader in APP research and development, as the roles originated there and the most peer-reviewed publications for nurses, APNs, and PAs are US-based.

Delamaire & Lafortune’s OECD Health Working Paper 54 (2010) states that: “Developing new and more advanced roles for nurses could improve access to care in the face of a limited or diminishing supply of doctors. It might also contain costs by delegating tasks away from more expensive doctors” [9] (p. 4). Furthermore, the OECD determined that the US and Canada would be the benchmarks for the development of APN professionals internationally. Much of our evaluation is based on APN roles, as PAs have developed in fewer countries (6 to date) and have fewer peer-review journals and evidence-based publications.

CNMs, although fully recognized in Israel, still have limited scopes of practice as compared to international standards. For example, most CNMs in the United States (US) are community-based and provide complete women’s healthcare including contraception and gynecological care. US-based CNMs are educated in a similar manner to other APPs, through graduate academic programs, yet Israeli nurse midwives are not required to have graduate-level education and work predominantly in hospital delivery units. They are required, however, to attain an additional license beyond a registered nurse (RN) license. CNMs have been successfully integrated and accepted into the mainstream Israeli healthcare system [53, 54].

Another type of APN role is the clinical nurse specialist (CNS). As this term is used internationally, CNSs do not *primarily* provide direct care, and do not usually have a license beyond the RN license [55]. CNSs are therefore not usually utilized for physician substitution, but instead focus on research, education and disease-based expertise [56, 57]. To further distinguish between the roles, CNSs enhance nursing practice for *nurses*, while other APP roles focus on direct patient care and supplementation or substitution for physicians. CNMs and CNSs are not included in our assessment of APP potential in Israel because of the existing widespread acceptance of midwives in Israel and CNS non-contribution towards physician supplementation.

Linguistically, due to a lack of translation of “nurse practitioner” into Hebrew (the primary language in Israel), the Hebrew term currently employed for the Israeli NP can be translated as "clinical nurse specialist". The scope of practice for the new Israeli role, which includes an emphasis on direct patient care, is closer to the international description of NPs rather than CNSs. Henceforth, we will refer to the Israeli model as NPs. In addition, titles “nurse”, “clinical nurse specialist”, “nurse expert”, and “nurse practitioner” are not protected titles in Israel, one may therefore find a range of nursing provider titles in various settings. This often leads to role confusion, especially as some people with “nurse” job titles are actually paramedics or students.

## Roles, education, and impact of advanced practice providers

### Advanced practice nurses

APN professions were established based on the needs of the medical system in times of war and physician shortage, and societal need in times of economic instability. Throughout the world, APNs have worked as non-physician health-care providers, effectively reducing physician shortages. There is a large body of evidence suggesting APNs provide effective, safe, equivalent, quality care in a wide variety of fields [11, 16].

According to the International Council of Nurses (ICN) and the International Federation of Nurse Anesthetists, there are currently four widely accepted APN professions including NPs, CRNAs, CNMs, and CNSs [58, 59]. The specific credentialing processes, roles, and practice of APNs are customized to each state and country. A master's degree in nursing is required or recommended in the majority of developed countries, excluding nurse midwives, and the American Association of Colleges of Nursing (AACN) has established a goal in the US of requiring doctoral level studies for new entry-level APNs [50, 51, 60, 61].

Notwithstanding the variability of preparation level, analogous themes are found in APN professional roles world-wide. Mantzoukas & Watkinson (2007) performed an international evaluation of the literature and identified 7 common, generic themes in APN roles internationally, as well as a common professional goal. Generic features include use of knowledge in practice, critical thinking/ analytic skills, clinical judgment and decision making skills, professional leadership and clinical inquiry, coaching and mentoring skills, research skills, and changing or improving clinical practice (p.32). In their assessment, the common professional goal was the attainment of professional autonomy [62].

**Fig. 1:**
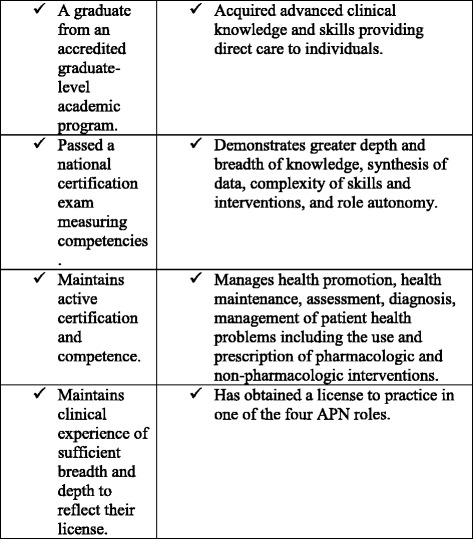
APRN definition - Consensus Model. Source: [64] (p.7-8)

The U.S. National Council of State Boards of Nursing defines Advanced Practice Nurses (APNs) as “registered nurses educated at Masters or post-Masters level [practicing] in a specific role and patient population. APRNs [Advanced Practice Registered Nurses, an alternative name to APNs] are prepared by education and certification to assess, diagnose, and manage patient problems, order diagnostic tests, and prescribe medications” [63]. The American Association of Colleges of Nursing defines APNs as nurses who meet specific criteria noted in Fig. 1 [64]. Due to the historic diversity in academic standards for APN professions, the APRN Consensus Model was introduced in the US as a “gold standard” for APN education. The core principles of standardized education and credentialing include requirements for three core courses: Advanced pathophysiology, advanced pharmacology, and advanced physical assessment [64].

APNs must specialize in at least one of six population foci: family/individual across the lifespan, adult-gerontology, pediatrics, neonatal, women’s health/gender-related or psychiatric/mental health [64] (p. 6). Sub-specialization occurs in the clinical setting, and promotes flexible career movement. For example, a family nurse practitioner can sub-specialize in geriatrics or pain management [64]. Global standardization of APN education is also underway through international European collaborations and the ICN [58].

### Nurse practitioners

Internationally, NPs are licensed clinicians that blend nursing and medicine into their clinical practice. Early in NP education, the university was established as the optimal academic setting. By 1989, 90 % of US-based NP programs were at the graduate level as a Master’s or post-Master’s certificate [65]. Each academic program requires hundreds of clinical practicum hours, beyond the clinical hours required for the undergraduate nursing degree, which must be completed in addition to didactic classroom instruction. NPs must also maintain a RN license, pass national certification exams, and maintain an additional NP license. Recertification is obtained through proof of Continuing Medical Education (CME) credits and active clinical practice.

NP education is based on expanding nursing knowledge to include advanced pathophysiology, diagnosis of disease, disease management, pharmacology, leadership, health policy, and education while incorporating the nursing paradigms of growth and development, health promotion, and disease prevention. The NP therefore provides a unique dimension of healthcare for chronic, complex, and acute situations [66]. By incorporating education into patient visits and working with patients and families as partners in healthcare, outcomes of NP primary care of adults and children have been reported to be equal or better to care provided by physicians alone, with less patient healthcare utilization and a more cost-effective bottom line [16, 67]. As early as the 1970s, studies (including systematic reviews, randomized clinical trials, and descriptive studies) evaluating the safety, outcomes, patient satisfaction, and other indicators show that NPs provide at least equivalent, cost-effective care for equivalent patient health conditions [68]. In the US, 40 % of primary care practices employ NPs [69]. Community-based NPs working in primary care can be found in countries across five continents (North America, Europe, Asia, Africa, Oceania) and is rapidly spreading due to efficacy of their work, impact on healthcare access, their work in underserved regions, and improved patient satisfaction ratings [70].

### Certified registered nurse anesthetists

According to the American Association of Nurse Anesthetists, CRNAs are defined as certified and licensed medical professionals who administer every type of anesthesia in every setting where anesthesia is administered. CRNAs are the primary providers of anesthesia care in rural US, and the main providers of anesthesia in the US military. They provide anesthesia services for every type of surgery and invasive procedure. CRNAs, at a minimum, have a Master’s degree, and they practice with a high degree of autonomy. They are required to be RNs, have acute care experience prior to applying to CRNA programs, and must maintain certification and licensure as CRNAs. They are re-certified through proof of CMEs and professional practice requirements [71, 72].

Nurse anesthetists have worked for nearly 150 years, pre-dating anesthesiologists by decades [72]. In 1906, Alice Magaw published the first study of nurse anesthesiology, which reviewed 14,000 cases with no fatalities attributable to anesthesia, a profound statistic at that time [73]. More recently, a Center for Medical and Medicaid Services (CMS) landmark study of 500,000 patient chart reviews showed that CRNAs provide equivalent anesthesia services to anesthesiologists with no increased complications or deaths [74]. An economic assessment in 2010 showed that independent CRNAs provide significantly more cost-effective care than supervised CRNAs or anesthesiologists alone [75].

In a review of the literature (CINAHL, MEDLINE, SCOPUS, PUBMED, Google SCHOLAR) published in 1990–2014, we were unable to find negative research about CRNAs. In a large Cochrane comparative review of over 1.5 million anesthesia cases administered by anesthesiologists, independent non-physician anesthesia providers (NPAs) such as CRNAs, and supervised NPAs, no conclusions were drawn about any difference in morbidity or mortality between the groups reviewed [76–78]. The International Federation of Nurse Anesthetists, representing 40 country members, acknowledges country-based variability in the autonomy, scope of practice, supervisory requirements (if any), licensure, education, and regulation of CRNAs [59]. To date, there have been no descriptive studies found regarding levels of autonomy of nurse anesthetists per country (as per our literature review described above).

### Physician assistants

According to the American Academy of Physician Assistants (AAPA), PAs are defined as certified and licensed medical professionals who practice medicine on healthcare teams with physician supervision. PAs perform medical histories, physical exams, diagnose and treat illnesses, order and interpret tests, develop treatment plans, write prescriptions and other activities [79]. Additionally, PAs can work as first assistants in surgery and perform many procedures that were previously considered physician-exclusive. According to the AAPA, PA duties “depend on the setting in which they work, their level of experience, their specialty, [and] State laws” [79]. PAs are educated in medical model clinical graduate programs, and most have a master’s degree. Previous experience in healthcare and prerequisite courses similar to medical school entry are required for entry into most PA programs.

The PA profession developed in the 1960s when experienced, post-Korean War paramedics returned to the US and needed to find suitable employment [80]. The development of NP and PA professions were within the same time period, so underlying societal and economic factors were similar for both professions. The educational development of the PA began as a post-Bachelor’s certificate program, and was not originally based in academic study. Moreover, they were not originally considered “professionals” (i.e. not possessing a *unique* body of knowledge), but specifically physician-extenders, created by physicians themselves.

Nursing, in comparison, has been considered a profession for decades based on academic education and extensive research. Nurses holding PhDs and other doctoral degrees in nursing are highly regarded in the academic world. The NP role evolved as an advancement of the nursing profession. There are over a hundred journals dedicated to nursing and APN research. There are fewer than ten dedicated journals for PAs. The PA profession is evolving and now has a growing body of unique PA-based research. PAs with doctoral degrees remain a rarity, and much of their education is still provided by physicians.

In a study of 118 US acute care hospitals, PAs and NPs were found to have little difference in their respective roles and in reimbursement for services [81]. Likewise, a survey of 246 US-based trauma centers reported that 33 % are already utilizing NPs *and* PAs in their trauma service, while an additional 19 % plan to incorporate PA and NP surgical sub-specialties in the future [82].

### APP impact and recognition

NPs and PAs combined represent 29 % of primary care providers in the US [83]. As of 2010, 52 % of US-based NPs and 43.4 % of PAs were working in primary care, totaling over 86,000 primary care providers [12]. APPs, including PAs and NPs, are predicted to provide a larger share of US primary care with the implementation of the Patient Protection and Affordable Care Act (PPACA) [84, 85].

In Europe, where APPs have been introduced more recently (with the exception of Great Britain), many countries are struggling with gaps in primary care coverage. A study evaluating gaps in primary, preventative, and coordination of healthcare in eight European countries (Austria, Belgium, Spain, England, Finland. Germany, The Netherlands, and Wales), shows that despite physician reluctance to delegate patient care duties to NPs, Dutch models of NP-inclusive primary care have shown positive results. Primary care NPs improved clinical outcomes, self-management skills, quality of life factors, and patient compliance with health plan [86] (p. 82). Furthermore, the study suggests that while patient populations remain suspicious of economic-based motivations of changing healthcare paradigms, they have high levels of trust in nurses working in advanced practice (p. 82). A British study found that primary care NPs provided at least equivalent care to patients requiring “same day” visits for acute episodic conditions [15].

There is no consensus international standardization for APN role recognition and definition [50]. Although we found consistency in education, separation of the APN role from the RN, and ability to perform previously exclusively medical functions, there is wide variability by country in the actual function and independence of the APN, particularly the NP [61]. The ICN lists country-specific definitions for the advanced practice roles. Many international studies utilize the ICN definition of NPs or APNs [9, 70, 87, 88]. In other international literature, NPs are also linked with PAs [81–83, 89]. Both professions have been shown to contribute towards solving physician shortages [80, 90].

More than 100 countries now recognize and differentiate APNs from RNs. The PA profession is recognized in some form in 6 countries. The educational requirements for APN professions internationally include graduate education as a pre-requisite for practice. Globally, all APNs must have a local RN license as prerequisite for an APN license. The educational requirement for PAs as of 2014 includes a clinical graduate degree [79]. Table 2 is a comparative analysis of various APP roles, educational requirements, and the number of countries accepting the roles.

**Table 2:** Comparative analysis of various APP professional roles

APP profession	NP	CRNA	PA
Education	7–8 years	7–8 years	5–7
Recognized # of countries	50+ (have or in development)	107^*^	6
Professional scope of practice	Assessment, diagnosis, treatment, prescriptions, patient management, referrals	Administer full range of anesthesia services, pre and post-operative assessments, in every setting where anesthesia is available	Assessment, diagnosis, treatment, prescriptions, patient management, referrals
Independent practice?	Most (US)	Yes	No. Requires physician supervision.
# (US) professionals	>205,000	>50,000	>95,000
Impact (US)	900 million patient visits	Administer 34 million anesthetics annually (2012) or 65 % of all US anesthesia	Unavailable

*Note: Internet search conducted 9/22/13 shows updates to original report such as official job availability and new educational programs since 1996. This search shows an additional 8 countries with CRNAs or nurse anesthetists

Sources: [12, 16, 65, 79, 87, 91, 93, 137, 138]

Most countries reported that APNs practice autonomously (without physician supervision or co-signature) or in collaboration with physicians [58]. PAs generally require physician supervision. There are currently over 205,000 NPs [65], over 50,000 CRNAs [91], and over 95,000 PAs [92] reported in the US alone. In the US, NPs provide over 900 million patient visits annually [65]. CRNAs provide 65 % of all anesthesia services in the US, with over 40 million anesthetics provided annually [91, 93]. No data was available for number of PA visits provided.

To sum up, as seen in Table 2, scope of practice in each respective country has some similarities, and includes many essential, independent functions that were previously considered exclusively physician "scope of practice". Internationally, there is not necessarily a relationship between scope of practice and independence in practice. Even within the US, levels of independent practice varies by state [94]. The IOM landmark report “The Future of Nursing” recommends expanding APN practice and encouraging independent practice [11]. To date, 20 US states plus the District of Columbia allow for completely independent NP practice, 30 states have partial independence, often requiring a collaborative agreement with a medical provider [94].

## Safety and quality of APP care

APNs have been shown to provide high quality solutions to physician shortages in dozens of countries worldwide in virtually every field they enter [61]. O’Grady performed a comprehensive evaluation of the literature about the quality of care for different APNs. A study of over 400,000 anesthesia cases showed “no statistically significant different in the mortality rate for CRNAs and anesthesiologists working together versus working individually…. [or] between hospitals staffed by CRNAs (without anesthesiologists) versus hospitals in which anesthesiologists provided or directed the anesthesia care” [16] (p. 2.603–2.604).

In another study conducted in the US comparing NPs and physicians, “No differences were identified in patient outcomes such as health status; physiologic measures; satisfaction; and use of specialists, emergency room, or inpatient services… NP care and physician care was comparable” [95] (p. 2.605). A British randomized study showed no difference in health outcomes between NPs and physicians in acute episodic primary care [15] Likewise, PAs and NPs have comparable results to physicians and to each other [12, 81].

A review of literature (CINAHL, MEDLINE, SCOPUS, PUBMED, Google SCHOLAR) did not reveal significant negative results of NP practice, which concurs with the OECD report that notes that in its evaluation of APNs internationally, no negative studies were found regarding patient safety or outcomes [9] (p. 43). Malpractice rates have been considered a marker of quality and safety of care. A study showed, for example, that between 1991–2007, physician liability rates *decreased* when a NP or PA was introduced into the practice [89, 95]. A comparative national review of malpractice lawsuit rates between primary care physicians and primary care NPs shows that NP lifetime rates are 6.29 per 1000 NPs compared to 249.75 per 1000 physicians [95]. A 2012 US survey showed that 2 % of NPs were named as primary defendants in malpractice suits [65].

An older systematic review from 2002, compares quality of care factors (patient satisfaction, length of consultation, prescription rate, return consultation rate, and referral rate) between physicians and NPs. No patterns of differences were found between types of healthcare providers, except for higher levels of patient satisfaction with NP providers [96]. This was also confirmed in a British study [15]. As most of the research draws similar conclusions, recent NP studies have focused on other aspects of NP-based care and less on comparative quality reviews.

## Motivating factors to APP implementation internationally

The main motivating factors for recognizing, or developing APPs worldwide includes physician shortages/need for improved access or delivery (US, France, Belgium, Scotland, Switzerland), reduced residency hours (US, UK), shortages in underserved regions (US, Canada, Finland, Australia), and cost containment (Germany, Netherlands, UK, US) by delegating tasks to less expensive professionals [9, 70, 97]. Direct and indirect economic benefits have been reported when APNs are integrated into healthcare systems. Direct cost benefits include less expensive and shorter training time for APNs compared to physicians. Furthermore, in most countries APN salaries are less than physicians. The indirect costs savings are even more substantial, as evidence shows the NPs reduce unnecessary emergency room visits, 30-day readmission rate, and waiting time for healthcare visits [98, 99]. A comparison of unit-based NPs and medical residents showed equivalent care, with a reduction in hospitalization days [100]. In current international trends of reduced residency hours, quality of patient care has not been reduced when NPs replaced residents [101]. The ability for APPs to get reimbursed for services is a significant factor in the success of their respective professions internationally, as their ability to get direct insurance reimbursements is another benefit from their services [102].

A Swiss meta-analysis shows that chronic disease management is improved or equivalent with NP care when compared to traditional physician care [10], likely due to nursing emphasis on health promotion and patient education. A 2005 European Cochrane review showed that compared to physicians, APNs provide equivalent quality care, with the same health outcomes, process of care, and utilization of resources at the same cost (prescriptions, medical tests, and 30-day readmission rates) [13]. A pilot study in France showed that APN care enhanced health outcomes in diabetic patients without increased cost [103]. While many of the studies appear cost-neutral, when adding the impact of lower education costs and lower salaries for APNs, APN care is more cost effective overall. In general, the focus of most comparative studies has been to show similar health outcomes, quality of care factors, and prescriptions/referral rates with less studies focused on cost.

## Barriers to APP implementation internationally

APP role implementation has historically faced challenges since the founding of these professions in the 19^th^ and 20^th^ centuries. Even though studies as early as 1907 showed safe anesthesia administration by nurse anesthetists [73], legislation to license CRNAs was repeatedly blocked by physician anesthesiologists [104, 105]. Historical international barriers to APN role implementation have included lack of educational standardization, lack of widely accessible education programs, lack of understanding of roles, perceived focus on a medical model of healthcare provision, the historical political strength of the medical profession, and credentialing issues [70, 85, 88]. Finally, there is a lack of title/ designation protection or title consistency internationally. Thirteen names for NP or APN nurse clinician equivalents were found, making comparisons and research more challenging [70, 87].

PAs have likewise faced similar barriers including territorialism by other health professionals, educational isolation, lack of understanding about professional value, resistance from physicians, and the variability of licensing restrictions [105, 106]. PAs also do not have a standardized international definition or title as evident in South Africa where they are known as “clinical associates” and have no independent functions [107].

Internationally, PAs and NPs collectively experience barriers in facility-specific policies, financial barriers, and resistance from medical staff [90]. There are noted faculty deficits in nursing, NP, and PA programs which create obstacles to increasing the availability of academic programs [108, 109]. Physician concerns with APN practice development include feeling threatened about their job security and financial viability [110, 111]. In many countries, physicians have also voiced concerns about clinical competencies of APNs [110], even though these concerns have been dispelled by decades of consistent evidence-based research showing excellence in quality of care [96, 101, 112-113].

## Status of APPs in Israel

The Israeli MOH recently released a description of the Israeli NP, which is similar to US and international definitions of NPs [17, 18], yet with some specific differences:

1. Israeli NPs are restricted in prescribing privileges and independent practice.
2. Israeli NP programs focus on institution-based practice.
3. NP education is delivered directly through the MOH.
4. NP education is a post-Master’s certificate with 6 months of part-time didactic education followed by a residency.
5. Israeli NP programs are currently subspecialty-specific, as guided by physician shortages in the field, limiting graduates’ professional mobility.

In contradistinction to current practices in *developed* countries, Israel’s NP specifications listed above reflected dated practices. Short, non-academic, and government exclusive programs continue to be used in developing countries with fewer resources [61]. Decades of evidence have galvanized regulations in favor of academic-level preparation, generalized studies as described by the APRN Consensus Model, and more independent practice. In the US, no evidence exists indicating that NPs from more restricting states are of different quality than in unrestricted states, but NP cost effectiveness is inversely related to such restrictions [11, 94, 114].

Over the last few years, two MOH sponsored NP courses were completed in geriatrics and palliative care, graduating several dozen Israeli NPs. In 2015, the Israeli MOH granted “diabetes NP” title and status to several expert diabetes care nurses, but MOH documents do not indicate that any formal NP training was provided [115]. Several more hospital-based specialty courses including surgical, neonatal ICU, pain management, are in planning stages with two courses being offered in late 2015. Thus far, to our knowledge, there are no efforts underway towards the establishment of any community-based or generalist/generic NP programs.

Each Israeli NP program thus far was designed by MOH committees, courses were/are provided by the MOH, and all licensing and certification is regulated by the MOH. NP education is currently not regulated by the Council for Higher Education and not provided by universities. Prerequisites for acceptance into NP courses included a Master’s degree, post-basic certification, and years of experience in the selected field [18, 88]. Courses consist of ~6 months of part-time classroom study followed by a physician-supervised clinical internship. As a comparison, the original US-based NP certificate programs circa 1965–1970 consisted of 12–18 months of study, and were likewise government-funded programs [116, 117].

A 2010 report on the state of Israeli medical and nursing manpower showed that trends were leading to severe professional shortages [21]. As a result, a MOH committee for “physician assistant” was formed. The MOH committee recognized the need for both NP and PA professions in Israel [46]. The committee also recognized the need for non-physician anesthesia providers or assistants, but few conclusions were drawn, and no progress has been publicized in regard to CRNAs. In 2015, the Director General of the MOH released a preliminary description of PA in Israel. According to the published circular, Israeli PAs will have mobility between hospital and community settings [118]. To our knowledge, no regulation or bylaws have been made public regarding the legalization of the Israeli PA profession.

In summary, in the last 50 years APP professions have developed internationally due to substantial evidence-based literature supporting the efficacy, safety, cost-effectiveness, and quality of care of these professions. World-wide trends towards standardizing and generalizing education, requiring academic education, and advancing the independence of APPs. Although the Israeli MOH recognizes the need for NPs and PAs, there is deviance from international standards. The Israeli MOH has provided a description of the future PA profession, but no further progress has been made to our knowledge. Finally, no progress has been made towards defining or developing programs for CRNAs in Israel.

## Discussion

### Health policy implication: The future of APPs in Israel

This paper has reviewed the acute problem of physician and nursing shortages and the role of APPs in addressing these shortages internationally. In Israel, one benefit to APP utilization would be the ability to provide healthcare to underserved areas, such as the geographic periphery. Areas of specialization-specific shortages such as trauma centers, understaffed hospital units, surgical suites, and primary care settings would likewise benefit from APPs [28, 46]. APNs have been providing primary care in remote regions worldwide, including Canada, Australia, and the US, even before the professions were officially recognized [119, 120]. International research demonstrates that APNs can alleviate both geographic and specialization-based physician shortages, expand healthcare accessibility, and improve the quality of healthcare provision [11, 16]. PAs are also an evidence-based viable solution to physician shortages [80, 121].

The anesthesiology shortages are worsening as medical students are less interested in entering the field [122]. The larger impact of this provider gap affects many areas of healthcare in Israel. For example, a patient requiring hip surgery who must wait many weeks for surgery may have higher levels of mortality, morbidity, and require more extensive rehabilitation due to postponed treatment [123].

We have shown that the literature supports the notion that adding APNs to healthcare teams could reduce the delays to point-of-care and extend the effective reach of Israeli physicians. Physician time may be better utilized by focusing on complex patient care, leaving routine healthcare to NPs. Additionally, evidence-based research demonstrates that APNs provide value-added services (i.e. supplemental services) beyond filling existing gaps in physician staffing (i.e. substitution services), since they are experts in health promotion, patient education, and disease prevention, and are considered more user-friendly according to patient satisfaction surveys [9, 124, 125]. NPs also spend more time explaining health conditions thus, patient self-management is improved and patient satisfaction rates are higher [15, 86]. Thus, APNs add a dimension of care otherwise not provided.

Even though many countries have determined that APP roles are indispensable within their healthcare systems, barriers remain in the recognition and integration of these roles in the Israeli healthcare system. One of the most significant barriers, is a lack of title protection for the term “nurse”, “nurse practitioner”, “nurse expert”, “nurse specialist” and a clear Hebrew translation of NP or APN. Israeli healthcare professionals, especially those who trained abroad, oftentimes utilize the English term “nurse practitioner”. Few Israeli-trained healthcare providers have an accurate understanding of what an NP or APN is. To further complicate matters, the MOH Division of Nursing created the term “clinical nurse specialist” and “expert nurse” to be used interchangeably for both NP and CNS roles in Israel [126]. This discrepancy in names has caused confusion. In Israel, for example, any nurse can be called a “specialist” by taking a post-basic course, yet being a “specialist” is not the same as an Israeli “clinical nurse specialist” [127]. Moreover, neither of these terms is protected or exclusive to specific role designations. World-wide, title protection and definition remains a pertinent issue.

A critical analysis of the 2013 MOH committee report on “Physician Assistant”, which documents policy development for APP professions, reveals several weaknesses. First, there were no APPs represented on the panel, nor were any consulted for expert opinion. Second, none of the numerous international APP organizations were consulted regarding professional designation, education, and scope of practice. Third, the MOH report was not data-based, nor based on scientific evidence from the existing body of literature regarding the integration and utilization of APPs globally. Fourth, the MOH panel recommendations for education standards of Israel’s proposed APPs were not based on international criteria and standards. For example, the panel suggested that paramedics could be educated to become anesthesiology “assistants” and “physician assistants” with one year of training [46]. In contradistinction, current international education standards for APPs require or recommend a minimum of a clinical master’s degree, as seen in Table 2. Accordingly, the validity of the findings and the recommendations of the report are questionable.

While the MOH report recognizes the need for APPs [46], only small groups of nurses have received NP education to date. Likewise, courses offered are not consistent in length and breadth to current NP education in other developed countries. In fact, considering the short, part-time course, MOH programs appear more congruent with NP education in *developing* countries where resources are more limited [61]. The MOH has adopted a 50-year-old model of NP education rather than using a contemporary, mature, evidence-based model, such as the APRN Consensus Model, which is accepted throughout the world and supported by the OECD and other international bodies. The rationale for this is unclear.

The APRN Consensus Model is predicated on the medical model of education: Generalist NP education followed by clinical specialization. Advanced specialization is attained after meeting post-generalist qualification levels. Physicians choosing to sub-specialize do so through clinical residencies and fellowships. Among NPs, for example, an experienced oncology nurse must earn a generalist NP degree in family, adult, or pediatric medicine. After graduation and board certification, one can choose to do a specialty clinical rotation in oncology before working as an oncology NP independently.

Before the introduction of the APRN Consensus Model in the US, there was academic variation with a trend towards NP sub-specialization, leading to differences in standards, education, scope of practice, and regulation by school, region, state, and specialty. Since the implementation of the Consensus Model and similar models internationally, APN education, regulation, and practice has become more uniform. There are many advantages to allowing more generalized and uniform NP education including job flexibility, improved community-hospital care coordination, and lowered APN education expenses. Israel could benefit from following an established model, such as the APRN Consensus Model, which would give structure and guidance for the new NP profession.

Unlike the APRN Consensus Model, the Israeli NP model consists of independently developed sub-specialty clinical programs described above. Consequently, Israeli NP sub-specialists cannot work in other clinical areas and there is little job flexibility. Thus Israeli NPs have a limited scope of practice and limited professional mobility compared to their international colleagues.

The determining factor regarding international utilization of NP models similar to the Israeli one is whether it is a developing or developed country, i.e. whether or not the country has the education and resources to support the new role adequately, as recommended by the ICN standards for APNs. Israel is universally considered a developed country and therefore appropriate resources must be allocated to ensure appropriate education and standards for this new role. Singapore, which developed the APN profession in recent years requires a Master’s Degree, 2 years of advanced education, hundreds of clinical hours, and nursing-exclusive APN educators [128]. Israel’s NP education is primarily provided by physicians and with standards that differ from international standards. For example, the “advanced physical assessment” course for the current surgical NP students was scheduled to be a 1.5-h long lecture. The APRN Consensus Model values advanced physical assessment as part of a core curriculum – as an individual semester-long course with clinical components [64].

In Israel, the critical shortage of nurses has an impact on the advancement of nursing. Ganz and Toren (2014) report that nurses in Israel describe poor work environments attributed to poor staffing and resource allocation [37]. In fact, a major critique for developing APNs in Israel has been that the nursing pool from whom APNs can be recruited and trained, is relatively small and may be negatively impacted through APN recruitment [46, 129]. The OECD [9] disagrees with this sentiment, stating that “the development of more advanced roles for nurses is often seen as a way to *increase* the attractiveness of the nursing profession and retention rates by enhancing career prospects [emphasis added]” (p. 9).

In the OECD survey results, Poland, Cyprus, Ireland, and the Czech Republic agreed that APN role development increased the recruitment and retention of nursing professionals. Additionally, “improving career prospects for nurses” may result in less emigration of these healthcare professionals to more lucrative careers abroad [9] (p. 19), thus decreasing the international “brain drain” phenomenon. A review of nursing retention strategies reinforced the concepts that encouraging nursing autonomy and promoting independent practitioners were key factors in improving clinical practice environments and retention [130] (p. 88).

A recent OECD report shows that from 2000–2013 Israeli nursing ratios decreased while physician ratios remained unchanged [31]. This data shows that the national plan to address physician shortages has been partially successful thus far. The lack of a national plan to improve nursing shortages is likewise reflected in the deteriorating numbers.

While hundreds of evidence-based studies show that APPs provide high quality, equivalent healthcare when practicing independently, significant barriers continue to exist internationally, and even within different US states [94]. Although more independent practice is evolving, this is a process that will likely take several years. In assessing historical integration processes from other countries, independent practice evolved over years with the maturing relationship between physicians and APPs. Maturity appears to be an important factor in determining the success of new APP roles.

Tens of foreign-trained and licensed APPs who have immigrated to Israel, are currently unable to practice. Foreign-trained APPs represent a significant, underutilized resource in Israel. Many of these healthcare professionals continue to work abroad in order to maintain their licenses and certifications, and to provide incomes for their families. This illustrates another example of the “brain drain” phenomenon reported in the literature [4]. Many other foreign-trained APPs living in Israel are underemployed or begin new professions.

There is tremendous potential for APP utilization in Israel. Many gaps in the healthcare system can be filled by APPs. Nurturing these professions during their infancy will ensure their successful integration into the Israeli healthcare system. It is essential to not only review Israeli health policy barriers and needs, but to also understand international historical trends with regards to successful integration of these professions. Israel can proactively smooth the way for APPs by anticipating concerns and developing policies to support both professionals and stakeholders in moving Israeli healthcare into the future.

### Recommendations

APNs promotes another level of differentiated nursing practice; advanced practice. In creating new position structures, there are opportunities for professional growth, increased salaries, and improved professional image; key factors in Israeli nursing recruitment [131, 132]. We believe that the addition of APNs to the nursing profession raises the economic and practice ceiling for nurses, which has the potential to attract more recruits to the larger nursing profession. In addition, as a quarter of Israeli nurses are not currently working in the healthcare sector, improving the attraction to the profession may motivate some of these nurses to return to the healthcare workforce.

We advocate that there may be advantages to grouping the roles of APN and PA into a single title of APP because of similar academic training, international role definitions, similarities of policy and regulation, and barriers to practice. While this may not be advantageous in countries with established professional roles, it may be particularly beneficial in small healthcare systems such as in Israel, where nursing self-perception is sub-optimal [133] and professional recruitment is difficult [131]. The title is not meant to diminish the nursing profession in anyway, but to raise the professional and cultural ceiling by widening the scope of shared professional practice. This title is much preferred to the title of the MOH commission that utilized the terms “physician assistants” and physician extenders in reference to APNs [46].

There is precedent for the APP title, as similar groupings exist, such as the title of “non-physician provider” used in the US and elsewhere. APP as a title may be especially applicable in settings that struggle with role perception or understanding, such as acute care settings. In primary care settings, where the focus is more health promotion oriented, the nursing model and APN title may be more appropriately utilized. In the US, PAs and NPs collaborate and work together. Many hospital practice sites employ PAs and NPs, with comparable job descriptions, pay, and scope of practice [81]. Though the training of each profession may differ, the combined effect of using both professions may be advantageous [102].

If the Israeli NP model adapts a mature, standardized model such as the APRN Consensus Model, the return on investment for *generalized* NP education will be increased, both financially and clinically. The broader the model of NP education and role, the broader the effect Israeli NPs will have on healthcare delivery. Job flexibility will allow the Israeli NP to move to areas of need, rather than be stagnant in areas of training.

Since Israeli nurses have similar, unionized salaries in all work environments, nurses are unlikely to change jobs even when dissatisfied. Presently, there is little financial incentive for nurses to change jobs, and recruitment to areas of need is challenging. We recommend moving towards a model of offering financial incentives that has been successful thus far in improving physician recruitment to needed areas. Moreover, nursing should evolve towards free market wages to allow institutions to compensate nurses more freely, especially in areas of extreme shortage. This would also improve nursing work environment and patient safety, according to the reviewed literature [45].

As in all new projects, stakeholders must be identified before they can be engaged in understanding APP roles [88]. The MOH has not yet defined who those stakeholders are, and what the full extent of the APP roles will be. Furthermore, the MOH excluded vital stakeholders from the Committee for “Physician Assistant” [46], despite recommendations to do so from the 2010 physician and nursing manpower report [21]. For example, the Israeli Nursing Association (INA) was not part of the core committee for evaluating APP roles. The INA must be engaged in lobbying for the advancement of APN roles. Most importantly, the public, as the largest stakeholder in Israel, must be informed and educated about APPs and their successes internationally in order to promote confidence in the new healthcare providers.

Title protection and consensus is vital to ensure the security and quality control of the NP profession in Israel. It is crucial for the MOH to legally protect the title of “nurse” and “nurse practitioner” and eliminate all similar titles from national medical lexis. This will avoid role confusion and ensure that people have the stated qualifications when using the protected titles.

In Israel, general nursing education is delivered by academic institutions while the MOH Division of Nursing and the Council of Higher Education sets guidelines and monitors programs. Combining the role of education provider and regulator for NP programs, limits the MOH’s ability to objectively evaluate itself and its programs, and constitutes a conflict of interest. In the majority of countries, the providers and regulators of nursing education and clinical guidelines are rarely performed by the same organization. Moreover, in the current state, there is less opportunity to educate larger numbers of NPs, as the MOH has limited resources. Accordingly, most APP programs in developed countries are based in graduate academic settings.

We therefore recommend that NP education in Israel be consistent with international standards of developed countries. The MOH should be responsible for transparency in setting academic and clinical standards for NPs to qualify for licensure. The MOH must maintain its objective role as regulator for NP standards of practice, and overseer of NP licensure and certification. NP education must be delivered through graduate academic clinical programs in institutions offering a master’s degree in nursing. The Council for Higher Education should monitor NP academic program delivery, as they do for all academic degrees.

Foreign-trained APNs with years of experience could bring maturity into a profession currently in its infancy in Israel. We believe that these professionals should be integrated into the healthcare system, by facilitating a pathway for their recognition and licensure. Foreign-trained APNs can be utilized as role models and may expedite the integration of NPs into healthcare settings. Benner’s Novice to Expert theory of nursing supports the notion that experts develop over time with experience and education [134]. Expert nurses who take NP coursework - become *novice* NPs. Expert nurses are unable to teach NP clinical skills. Likewise, physicians, who are presently the primary educators of NPs in Israel, are unfamiliar with expert NP practice and roles. They are unable to teach NP role integration and the nursing-medical bridging required to become a proficient NP. Overall, clinical maturity of NPs in Israel is limited by not taking advantage of expert mentors such as experienced, foreign-trained NPs who already live in Israel.

Although a long-term goal for APP independence in Israel should exist, the reality is that a team approach linking physicians and APPs may be a more realistic and culturally competent short-term solution. Introducing APPs as team members is less threatening to physicians who are used to being the sole medical care providers. NPs working in teams may help reduce barriers to practice, improve designation of new professional boundaries, and facilitate the integration of new professions into healthcare settings [135]. In the Netherlands, a deliberately slow integration of NPs into primary care has led to physician support for primary care NPs. The evidence supported the shared team-based model with regard to beneficial interdisciplinary processes and patient outcomes [86] (p. 84).

The extreme shortage of anesthesiology services in Israel impacts many fields and has a high cost in terms of patient morbidity and mortality due to delayed surgeries. Considering the data reviewed, establishing the CRNA in Israel would be a relatively fast (2–3 years) way of improving accessibility to anesthesia services, especially in the periphery. CRNAs, as opposed to paramedics with a 1-year course (suggested by the 2013 MOH committee) [46], have the strongest, time-tested evidence-based practice in terms of patient safety, quality of care, longevity, and ability to work independently. CRNAs have been shown internationally to provide equivalent care to anesthesiologists. CRNAs in Israel will not replace anesthesiologists, but will extend the reach of anesthesiologists in Israel. One anesthesiologist could monitor several surgeries at once while CRNAs provide anesthesia services in the operating room, seeking physician consultation as needed. We therefore recommend that the MOH prioritize CRNA programs to improve patient accessibility to safe, quality anesthesia services as soon as possible.

Finally, the enormity of the worsening nursing shortage in Israel must be mitigated through an elimination of bureaucratic barriers, increasing the number of nurses in the healthcare workforce, and an improvement of the public’s professional nursing image, which NP development may help [131, 132]. The integration of APPs into the healthcare workforce is the most expeditious, evidence-based approach of alleviating the physician shortage in Israel. Since the NP role has already been initiated into the healthcare system, albeit on a very limited scale, it is reasonable to suggest that these programs continue to expand, become transparent, shift to publicly available academic programs, and effectively include all stakeholders and foreign-trained NPs in the rapid advancement of this profession.

### Areas for future research

Our investigation notes a gap in the literature regarding international comparative reports on APP professions. In a global world, with emigration being a commonplace phenomenon, increasing the clarity by understanding the processes and challenges by which healthcare professionals are recognized, educated, and integrated into their countries of choice may help to expedite the process towards licensing and gainful employment. No studies have evaluated foreign-trained APPs living in Israel. Future pilot studies could be initiated to assess the integration of foreign-trained APP immigrants to Israel. Other studies should include interviewing key policy makers as to their general attitudes towards APP integration into the Israeli healthcare system. It would also be useful to study how the training of APPs is financed in other countries and to consider the pros and cons of various options for how such training could be financed in Israel.

The economic impact of NPs in Israel also cannot be assessed as there are too few NPs currently working in Israel. In the US, NPs, PAs and CRNAs were found to be cost-effective providers, but this may be different in Israel where socialized medicine has produced lower overall salaries in the healthcare field. Future study of the health economics of NPs and other APPs in Israel is essential. Finally, patient health scores for those with chronic diseases could be assessed before and after the introduction of APPs into specific clinical settings; similarly, patient, nurse, and physician satisfaction of care could be reported as the new role is introduced.

As international economic comparative studies between APNs, PAs, and physicians may not apply to all countries – in depth analyses are needed in Israel to evaluate the cost-effectiveness of the new professions, including direct and indirect comparative cost. This is particularly vital as Israel, like all countries, must justify spending and budgets in healthcare and seek more resource-friendly options.

Even though international literature has shown consistent positive comparative quality and outcome results between APNs and physicians, these studies must be repeated in Israel to show the effectiveness of NP delivery of quality care. These studies will also strengthen the argument for supporting other APP professions and expanding current levels of practice.

### Limitations

Many European countries have started APN education programs in recent years [97], limiting the availability of research from those countries. Although we attempted to include as much relevant data from a wide range of countries, a majority of the literature originates from English-speaking countries, which has been acknowledged by other international researchers [9].

Authors respectfully acknowledge their status as Israeli RNs who remain unrecognized, foreign-trained, licensed, and certified nurse practitioners living in Israel.

## Abbreviations

ANPI: Association of Nurse Practitioners in Israel
APN: Advanced practice nurse
APP: Advanced practice practitioner
APRN: Advanced practice registered nurse
NP: Nurse practitioner
MOH: (Israel) Ministry of Health
PA: Physician assistant
CRNA: Certified registered nurse anesthetist
CNM: Certified nurse midwife
CNS: Clinical nurse specialist
ICN: International Council of Nurses
AACN: American Association of Colleges of Nursing
US: United States of America
IOM: The Institute of Medicine of the National Academies
OECD: Organisation for Economic Cooperation and Development
RN: Registered nurse
MD: Medical doctor
NCLEX-RN: National Council Licensure Examination for Registered Nurses
USMLE: United States Medical Licensing Examination
AANP: American Association of Nurse Practitioners
AAPA: American Association of Physician Assistants
AANA: American Association of Nurse Anesthetists
CME: Continuing medical education
ACA: Patient Protection and Affordable Care Act
INA: Israel Nurses Association
NPA: Non-physician anesthesia providers

## Footnotes

1 This will be the case even though many physicians continue to work past the age of 67.

2 Incentivizing foreign physicians to immigrate to Israel has included financial incentives for physicians moving to Israel [44] and easing the lengthy bureaucratic processes.
